# Backache from Weak Heart

**Published:** 1895-08

**Authors:** Charles G. Stockton

**Affiliations:** Professor of the principles and practice of medicine and clinical medicine. Medical Department, University of Buffalo; Physician to the Buffalo General Hospital.


### BACKACHE FROM WEAK HEART.

By CHARLES G. STOCKTON, M. D.,

Professor of the principles and practice of medicine and clinical medicine, Medical Department, University of Buffalo; Physician to the Buffalo General Hospital.

AN INTERESTING condition that seems, for the most part, to have escaped notice is represented by a long series of cases that have appeared in the wards of the Buffalo General Hospital, besides a collection that has occurred in the practice of the writer.

The clinical picture consists of an extremely feeble heart, apparently beginning without dilatation, or the usual gross lesions that are regarded as ordinary causes of cardiac enfeeblement, associated with venous distention, poor capillary circulation, cyanosed extremities, occasionally slight edema of the ankles, headache, insomnia, disturbed digestion, constipation, scant, dense and hyperlithuric urine, usually very acid in reaction, and last,—the symptom that usually attracts most attention,—pain in the back. This pain appears in the lumbar region. It is not like lumbago, but resembles more the aching sometimes observed in those who have very acid urine accompanied by uric acid sand.

In the condition in question the pain is believed not to depend upon the state of the urine alone, since it has been known to continue when the urine had been made neutral in reaction by the use of alkalies. It probably in part results from the condition of the urine, and in part from the slowing of the blood current through the kidneys and the unusual venous pressure. The latter factor is believed to be the more important, since by correcting the circulation the pain may be relieved promptly, yet such relief is not obtained by other methods of treatment.

The condition of the heart deserves further consideration. While in the beginning the heart is not found appreciably enlarged, it dilates progressively if the patient pursues his active life and does not receive adequate treatment. This may be determined by the increase of precordial dulness and by the appearance of valvular insufficiency. When this stage of the affection is reached the patient usually suffers from headache, dizziness and sometimes faintness. The heart can be made to assume its normal size and valvular insufficiencies disappear under appropriate management.

In my experience the majority of these cases occurs in men engaged in laborious work out of doors. So frequently have the lumbermen and woodsmen of North Pennsylvania appeared in the wards of the hospital suffering from this cardiac enfeeblement that, in that institution, it has come to be known as the "Pennsylvania heart." Apparently the condition has supervened upon some transient cardiac depression in those who, unacquainted with the symptoms and not appreciating the defect in circulation, continue with their usual habits.

I have no recollection of seeing these cases previous to 1889, and it has occurred to me that this may be explained by the severe epidemic of influenza that prevailed throughout this part of the country in that year. Exceptionally, a patient appears who denies having had influenza, but the vast majority describe a sharp attack and admit not having felt quite the same since.

While suggesting that the state above may arise from influenza, it is not intended to convey the idea that influenza alone is responsible for it. Not infrequently the same condition is seen to follow typhoid fever. Fortunately, the treatment is successful when it can be properly carried out. First and foremost, it means complete rest in bed, to which should be added vigorous massage treatment daily and general faradization. The use of the alternating hot and cold spray or douche is of service and, as for medicine, nothing has proved so serviceable as the administration of large doses of nux vomica. The tincture is preferred, and the dose should be increased drop by drop until its physiological effects are observed, and these may not be seen until the patient takes from 50 to 100 drops three times a day. Digitalis is at times necessary and it may be advisable to combine this drug with nitro-glycerine or belladonna, or both these.

Months sometimes elapse before the patient is able to resume the upright position for any length of time. The backache, however, disappears immediately. The condition is liable to relapse, and I am sure that many people have become disabled by the want of a proper understanding of the disease, or by the discontinuance of the treatment before the cure is cemented.

Finally, while a weak and dilated heart and a resulting depressed circulation is sometimes seen without backache, attention is particularly called to this symptom in connection with such conditions. When a laboring man complains of backache and feebleness, one is not likely to go wrong if he turns his attention immediately to the circulation for the explanation of his case.

436 Franklin Street.

